# Dataset of occurrence and incidence of pine processionary moth in Andalusia, south Spain

**DOI:** 10.3897/zookeys.852.28567

**Published:** 2019-06-05

**Authors:** Andrea Ros-Candeira, Antonio Jesús Pérez-Luque, María Suárez-Muñoz, Francisco Javier Bonet-García, José A. Hódar, Fernando Giménez de Azcárate, Elena Ortega-Díaz

**Affiliations:** 1 Laboratorio de Ecología (iEcolab), Instituto Interuniversitario de Investigación del Sistema Tierra en Andalucía (CEAMA), Universidad de Granada, Avenida del Mediterráneo s/n, 18006, Granada, Spain; 2 Grupo de Ecología Terrestre, Departamento de Ecología, Universidad de Granada, Facultad de Ciencias, Campus Fuentenueva s/n, 18071, Granada, Spain; 3 Departamento de Botánica, Ecología y Fisiología Vegetal, Área de Ecología, Universidad de Córdoba, Edificio Celestino Mutis (C-4), 14014 Córdoba, Spain; 4 Agencia de Medio Ambiente y Agua de Andalucía. Consejería de Medio Ambiente y Ordenación del Territorio, Junta de Andalucía, C/ Johan G. Gutenberg 1-Isla de la Cartuja, 41092, Sevilla, Spain; 5 Consejería de Medio Ambiente y Ordenación del Territorio, Junta de Andalucía, Casa Sundheim, Avda. Manuel Siurot 50, 41071, Sevilla, Spain

**Keywords:** Degree of defoliation, forest pest, monitoring, pine plantations, pine woodlands, sampling event, southern Iberian Peninsula, *
Thaumetopoea pityocampa
*

## Abstract

This dataset provides information about infestation caused by the pine processionary moth (*Thaumetopoeapityocampa* ([Denis & Schiffermüller], 1775)) in pure or mixed pine woodlands and plantations in Andalusia. It represents a long-term series (1993–2015) containing 81,908 records that describe the occurrence and incidence of this species. Data were collected within a monitoring programme known as COPLAS, developed by the Regional Ministry of Environment and Territorial Planning of the Andalusian Regional Government within the frame of the Plan de Lucha Integrada contra la Procesionaria del Pino (Plan for Integrated Control Against the Pine Processionary Moth).

In particular, this dataset includes 4,386 monitoring stands which, together with the campaign year, define the dataset events in Darwin Core Archive. Events are related with occurrence data which show if the species is present or absent. In turn, the event data have a measurement associated: degree of infestation.

## Rationale

Monitoring programmes are conducted in numerous countries and regions affected by this forest defoliator. The detection of this species is simple, since larvae nests are easily visible on affected trees and defoliation becomes obvious at a certain level of infestation. Thus, monitoring often consists on the assignment of infestation indexes to plots based on visual observation and following a discrete scale (see “Methods”).

**Figure 1.**
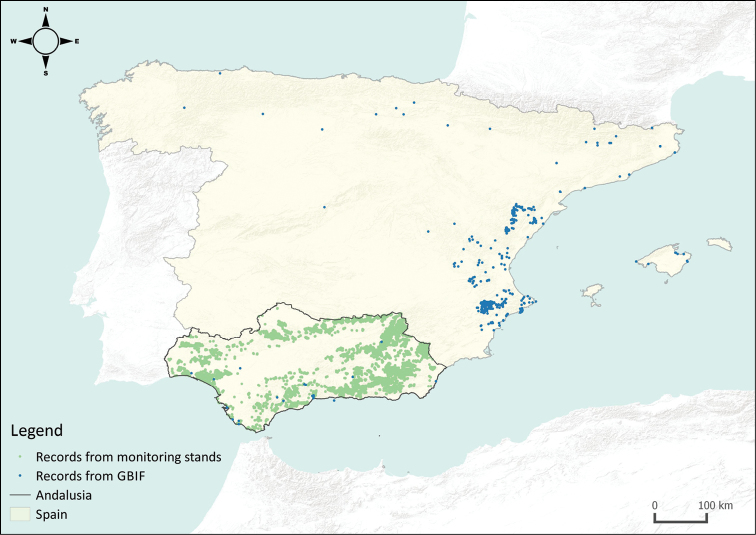
Distribution of *Thaumetopoeapityocampa* records from GBIF in Spain and records provided in this dataset. Records from GBIF were downloaded on 2018-03-16 using the R package “rgbif” (Chamberlain et al. 2016).

Unfortunately, those existing time series are rarely available for the scientific community. In the case of GBIF, the volume of data regarding this species is scarce (Fig. 1). In the current context of climate change, information about forest pests becomes important since pests can play a fundamental role affecting the physiology of forest ecosystems (Gracia et al. 2005). This data paper, therefore, constitutes an initial step towards the sharing of such time series, aiming to encourage studies using them, especially in relation to management decisions regarding forests phytosanitary status and ecological studies about population dynamics, as well as other research areas.

## Taxonomic coverage and ecological importance

The whole dataset includes 81,908 records that describe the occurrence of a single species: *Thaumetopoeapityocampa* ([Denis & Schiffermüller], 1775).

**Figure 2.**
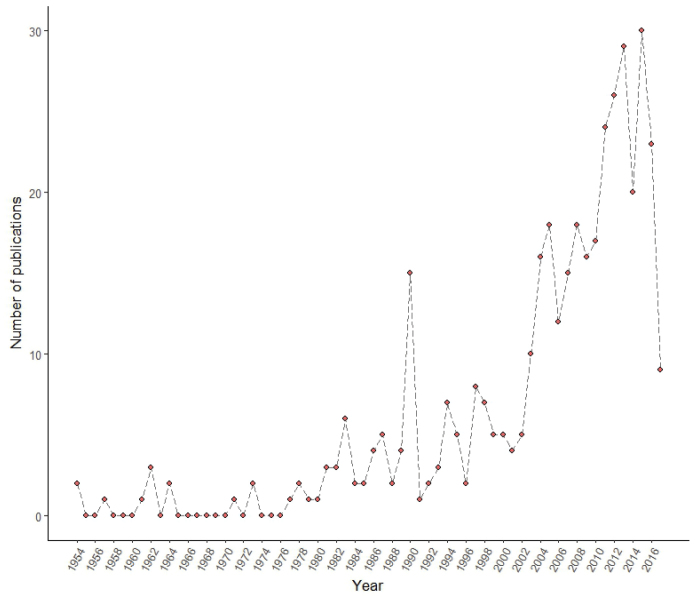
Number of publications per year about *Thaumetopoeapityocampa* in Web of Science (search date 2017-10-05) since the first publication registered.

The pine processionary moth is a well-known species, receiving a great attention by the scientific community, from medicine to ecology. In October 2017, Web of Science referred 400 publications mentioning this species, showing an increasing tendency in the last decades (Fig. 2; see Roques (2015) for a recent review on the taxonomy and biology of the genus). Due to its defoliating activity and to the allergic reactions it causes on animals and humans, scientists have studied this species in detail for a long time. Substantial efforts have been made to understand the life cycle, population dynamics, and main factors affecting this species, as well as the mechanisms involved in the urticating reaction caused by larval hairs. Moreover, control measures and management of affected forests are also important research lines.

In Andalusia, *Thaumetopoeapityocampa* is one of the species that causes the most extensive impact to the pine forests, either natural or planted. The typical distribution area of this species is conditioned by climate and associated with Mediterranean and circum-Mediterranean regions (Battisti et al. 2015), mainly feeding on the genus *Pinus*. This distribution is explained to a big extent by the minimum winter temperatures as the larval stage takes place during winter (Buffo et al. 2007; Démolin 1969; Hoch et al. 2009). Therefore, increasing winter temperatures favour this species and climate change is thus expected to increase the potential distribution of the species. Because of this, it has become a paradigmatic case study regarding the response of forest pests to climatic change (Netherer and Schopf 2010). Indeed, reports already show presence, outbreaks and potential shifts of the species at higher altitudes and latitudes than before (Battisti et al. 2005; Pimentel et al. 2011). It should be noted that *Thaumetopoeapityocampa* is not an alien or invasive species in Andalusia, being both the moth and the host pine species autochthonous from the region. However, the combination of climate warming and man-made pine spreading has resulted in a very favourable situation for its expansion in its full distribution area, thus causing its usual consideration as a pest (e.g., EPPO 2004).

### Taxonomic ranks

**Kingdom**: Animalia

**Phylum**: Arthropoda

**Class**: Insecta

**Order**: Lepidoptera

**Family**: Notodontidae

**Genus**: Thaumetopoea

**Species**: *Thaumetopoeapityocampa* ([Denis & Schiffermüller], 1775)

**Common Name**: pine processionary moth

### Spatial coverage

**General spatial coverage**

Andalusia is located in Southern Spain and covers around 87,597 km². This is a region characterised by great climate variability. Though the majority of the surface is classified as Mediterranean climate type (Csa, according to Köppen’s classification) (AEMET 2011), there are other bioclimatic zones: subtropical (Mediterranean coast), oceanic (Atlantic coast), mountain (medium and high mountain areas in mountain ranges which reach 2000 m a.s.l.), subcontinental (Guadalquivir Valley and part of oriental Andalusia) and sub-desert (Southeast zone with coastal influence) (Junta de Andalucía 2014). The altitude ranges from sea level to Sierra Nevada summits, where the highest peak reaches 3481 m a.s.l.

The forest area, and specifically coniferous formations which encompass pine forests, has increased intensely during the second half of the 20^th^ century. Due to past reforestation projects (Gutiérrez-Hernández et al. 2016), its area has doubled in 51 years (1956–2007) (Muñoz-Rojas et al. 2011). The reasoning in these reforestation was their commercial value and the general economic interest underlying the National Afforestation Plan of the 40s (Junta de Andalucía 2011) and, in eastern Andalusia, the need to control soil erosion. This means that a high percentage of the pine woodlands in Andalusia are or were originally plantations. Muñoz-Rojas et al. (2011) in their research on land cover changes in Andalusia, highlight afforestation as the second major change because of the extension of the transformed area. The evolution of coniferous formations through time in thousands hectares has been as follows: in 1956 they covered 374.6, while in 1989 it was a surface of 764.1, in 1999 around 684.5 and in 2003 a total of 683.7 (Junta de Andalucía 2010).

In this scenario, *Thaumetopoeapityocampa* has found a large surface for its activity producing an impact on forests because of defoliation.

**Coordinates**

36°2’35.53’’N, 38°37’12.03’’N Latitude; 7°26’8.72’’W, 1°52’27.71’’W Longitude

### Temporal coverage

1993–2015

### Project details

**Project title**

Plan de Lucha Integrada contra la Procesionaria del Pino (Plan for Integrated Control Against the Pine Processionary Moth)

**Study area description**

The target ecosystem of the project is the great majority of Andalusian pure or mixed pine woodlands and plantations (Junta de Andalucía 2013). As noted above, as a result of intense reforestations in past decades the pine forest presence in Andalusia is extensive. The most common species are *Pinuspinea* L., *P.nigra* J.F. Arnold, *P.pinaster* Ait., *P.halepensis* Mill., *P.sylvestris* L., and occasionally P.sylvestrissubsp.nevadensis Christ (Junta de Andalucía 2010). In particular, the surface area covered by the monitoring stands included in this dataset is 7,717.6 km². It should be noted, however, that the present data only include forested areas. Pine processionary moth is also present in non-forested areas with occasional presence of isolated pines (gardens, roadsides, roundabouts), but for reasons of its scant surface they are not included in the monitoring programme.

**Design description**

Following European and national regulations regarding forest management and use of phytosanitary products, the Regional Ministry of Environment and Territorial Planning of Andalusian Regional Government implemented the Plan for Integrated Control Against the Pine Processionary Moth (hereafter referred as Plan), which began in 1991. This Plan came into place aiming to assess the evolution of this pest and defining preventive and control measures. As part of that, COPLAS monitoring programme was developed. It consisted of assessing annual defoliation caused by this species on pines and counting of nests through human observation. A survey system was designed to store the generated information, which is collected in a form for each monitoring stand. Within the plan, an important step after assessing the level of damage consists of issuing a proposal for actions or treatments and execute those control measures. According to the incidence of this species, the Plan considers different treatments to maintain the pine processionary moth population below a certain threshold, for example, from manual treatment of the nests or pheromone traps to spray treatments (Junta de Andalucía 2013).

The Plan was designed mainly from a preventive point of view, with the aim of controlling the population of the pest, but contemplating its dynamic character, incorporating large surfaces and new techniques over time. For instance, aerial spraying, a common procedure initiated a few years ago to reduce defoliation impact, is now almost completely forbidden according to EU guidelines (Directive 2019/128/EU). Treatments are, at the present moment, restricted to specific areas in which the pine processionary moth may have a direct impact on human or livestock.

## Methods

### Sampling description

For the monitoring programme COPLAS, pine forests were divided into monitoring stands according to administrative and environmental criteria defined in the Plan. Every year, these stands were visited at the end of the defoliating season (from end of winter to beginning of spring) and a defoliation degree was assigned to the plot based on observation of the stand as a whole.

The result was the production of a scale ranging from 0 to 5 which represents the degree of infestation by the pine processionary moth:

Degree 0: None or some very scattered nests are observed through the stand.
Degree 1: Some nests are observed at the stand edges, in clear areas as well as isolated trees.
Degree 2: Numerous nests at the edges of the stand, in clear areas and some in the middle of the stand.
Degree 3: Partial defoliation at the stand edges and isolated trees. Abundant nests in the middle of the stand.
Degree 4: Very strong defoliations oliations at the stand edges as well as isolated trees and partial defoliations in the rest of the stand.
Degree 5: Very strong defoliations throughout the stand.

Since this defoliation assessment was used to define further management measures, this initial assessment could be checked and further adjusted by a technician when plots were assigned a degree equal or higher than 3. Plots assigned with a degree of 2 were also checked if they were next to plots assigned with a degree of 3.

Every year, the Plan increased the area covered by the monitoring stands (Junta de Andalucía 2010), which are distributed throughout all the provinces of Andalusia.

### Step description

All data were stored in a normalised database (PostgreSQL) and incorporated into the Information System of Sierra Nevada Global-Change Observatory (Pérez-Pérez et al. 2012). Taxonomic and spatial validations were made on this database (see Quality control description). A custom-made SQL view of the database was performed to gather occurrence data associated to sampling event and other variables associated with occurrence data, specifically, degree of infestation.

The sampling event data, occurrence, and measurement data were accommodated to fulfil the Darwin Core Standard (Wieczorek et al. 2009; 2012). We used Darwin Core Archive Validator tool (http://tools.gbif.org/dwca-validator/) to check whether the dataset meets Darwin Core specifications. The Integrated Publishing Toolkit (IPT v2.0.5) (Robertson et al. 2014) of the Spanish node of the Global Biodiversity Information Facility (GBIF) (http://ipt.gbif.es) was used both to upload the Darwin Core Archive and to fill out the metadata.

The Darwin Core elements for the sampling event data included in the dataset are: eventID, modified, language, institutionCode, collectionCode, continent, country, countryCode, stateProvince, county, eventDate, habitat, minimumElevationInMeters, maximumElevationInMeters, decimalLatitude, decimalLongitude, geodeticDatum, coordinateUncertaintyInMeters, samplingProtocol, sampleSizeValue, sampleSizeUnit, footprintWKT. For the occurrence data the elements are: occurrenceID, catalogNumber, eventID, eventDate, basisOfRecord, scientificName, kingdom, phylum, class, order, family, genus, specificEpithet, scientificNameAuthorship, associatedTaxa, recordedBy, occurrenceStatus. For the measurement data, the Darwin Core elements included were: measurementID, eventID, measurementType, measurementValue, measurementUnit, measurementDeterminedBy, measurementDeterminedDate, measurementMethod.

**Figure 3.**
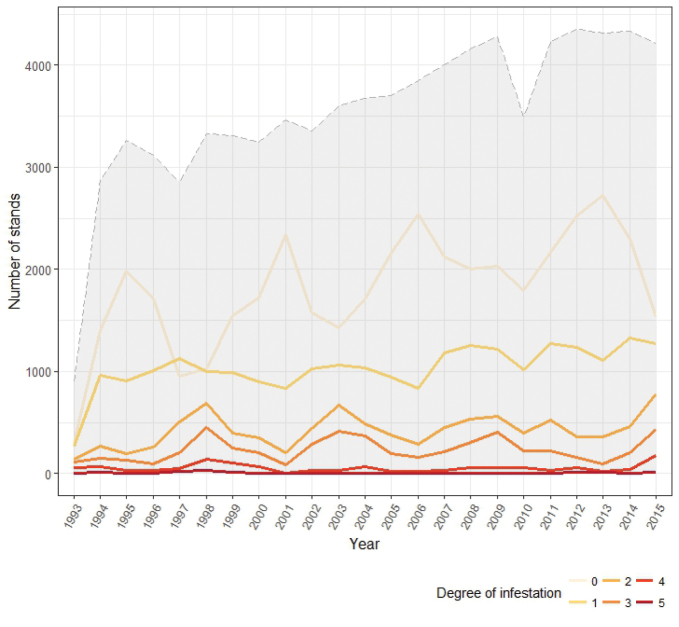
Number of monitoring stands per year according to defoliation degree. Gray area represents the total number of monitored stands per year.

### Quality control description

The scientific names were checked with databases of Catalogue of Life/Species 2000 (Roskov et al. 2018) and a recent review on the phylogeny of the genus *Thaumetopoea* (Basso et al. 2017). We also performed validation procedures (Chapman 2005a; 2005b) (geographic coordinate format, coordinates within provincial/county boundaries, absence of ASCII anomalous characters in the dataset) with Darwin Test (3.3) software (Pando et al. 2017).

**Dataset description**

**Object name**: Darwin Core Archive COPLAS: Dataset of occurrence and incidence of pine processionary moth in Andalusia (South Spain)

**Character encoding**: UTF-8

**Format name**: Darwin Core Archive Format (Wieczorek et al. 2009)

**Format version**: 1.0

**Distribution**: http://ipt.gbif.es/resource?r=coplas

**Publication date of data**: 2018-04-20

**Language**: English

**Licenses of use**: this dataset is licensed under the Creative Commons Attribution 4.0 International License (CC BY 4.0) https://creativecommons.org/licenses/by/4.0/

**Metadata language**: English

**Date of metadata creation**: 2018-04-20

**Hierarchy level**: Dataset
